# Introducing an Innovative Approach for Managing Proximal Non-Cavitated Carious Lesions in Juvenile Permanent Dentition: Combining Orthodontic Separators and Silver Fluoride Application

**DOI:** 10.3390/medicina59111892

**Published:** 2023-10-25

**Authors:** Eilaf E. A. Ahmed, Salma Al Nesser, Julian Schmoeckel

**Affiliations:** Department Preventive and Pediatric Dentistry, University Medicine Greifswald, Walther-Rathenau Straße 42a, 17475 Greifswald, Germany; eilafeltigani@gmail.com (E.E.A.A.); salma.alnesser93@gmail.com (S.A.N.)

**Keywords:** initial caries, silver fluoride, minimal invasive, caries diagnosis

## Abstract

Background and Objectives: The aim was to introduce an innovative, easy and cheap clinical approach for the control of multiple proximal non-cavitated lesions via the application of 38% silver fluoride after placement of orthodontic separators in the permanent dentition in high-caries-risk children. Materials and Methods: The case series describes the management of initial proximal carious lesions using silver fluoride (SF) products in the permanent dentition of two adolescent patients with prior proximal caries progression. Both presented with multiple asymptomatic carious lesions that were identified through the use of bitewing radiographs and classified according to the ADA proximal caries classification system. Using orthodontic separators prior to the planned application of SF, most of the surfaces could then be quickly directly examined to check for surface integrity. Follow-up examinations were conducted clinically and radiographically for at least one year to monitor lesion progression. Results: None of the 25 enamel lesions (E1/E2) exhibited signs of progression after single SF application, while 2 out of 11 dentine lesions (D1) showed progression and required restorative intervention. The progressed lesions potentially had non-cleansable micro-cavitations that were either clinically undetected or not fully reached with the micro-brush in SF application. Thus, this should have been repeated or combined with SF application via soaked superfloss to potentially achieve better results. Conclusions: Single application of 38% silver fluoride directly onto active enamel lesions in juvenile permanent teeth with the prior use of orthodontic separators combined with a caries-risk-specific prevention program appears to be highly effective and should be considered as a viable minimally invasive option for patients and clinicians due to its cost-effectiveness and time efficiency.

## 1. Introduction

Dental caries are dynamic, with an imbalance between remineralization and demineralization of the dental surface [1], and initial lesions are the earliest stage of this process. These develop when the enamel is demineralized under the subsurface beneath an intact surface layer of enamel [2]. These initial lesions can be reversed or arrested if remineralization outweighs the demineralization process, or transformed into a cavitation if demineralization outweighs the remineralization process [3].

Active initial caries appear clinically as chalky white lesions [4] and can be easily diagnosed on cleaned and dried occlusal or smooth surfaces. On the contrary, proximal surfaces have always been a challenge regarding the early diagnosis and consequently the control and management of the caries process. Clinical diagnosis within the area of contact points, limited salivary access and the inability to self-cleanse have shaped this challenge. In general, the caries process in the proximal area of the permanent dentition in low-caries-risk children is slow, especially in countries where a significant caries decline in children has been observed [5]. Nonetheless, caries incidence during adolescence is still high and mainly affects proximal surfaces.

Management of such lesions can range from improving oral hygiene practices and fluoride use to the application of proximal sealants, caries infiltration and traditional restorations [6]. Silver fluoride products (SF) offer a minimally invasive treatment option that has been shown to be effective in managing carious lesions in children and adults [7].

In addition to the gold-standard X-rays, alternative methods have been introduced over the last few decades for detecting caries without the use of radiation, such as optical coherence tomography (OCT) and near-infrared (NIR) and fiber-optic technology, which have contributed to the recently developed digital fiber optics (FOTI/DIFOTI) [8,9]. Using these methods together enables early diagnosis, potentially shifting the intervention from surgical to preventive treatment [3].

It is quite well known that SF provides significant chemical interference in the progression of caries and also has the ability to prevent the initiation of the caries process [10] for both primary and permanent teeth [11,12]. It has been recommended from in vitro studies that, especially in non-compliant patients, 38% SF be applied to remineralize incipient caries lesions of permanent teeth where aesthetics is not a concern [13], as it is capable of inducing/increasing enamel remineralization [7,14].

To the best of our knowledge, no clinical studies or case reports are available in the literature on SF treatment of multiple active non-cavitated proximal carious lesions in permanent dentition of children, despite the demonstrated high effectiveness of SF for all stages of carious lesions.

This case series was prepared following the CARE Guidelines [15], and aims to introduce an innovative, easy and cheap clinical approach for the control of multiple proximal non-cavitated lesions via the application of 38% silver fluoride after placement of orthodontic separators for direct clinical inspection and better access to the proximal lesion.

## 2. Case Presentations

**Case 1**

**Patient information**

In November 2019, a 13-year-old female patient in good health visited the Department of Preventive and Pediatric Dentistry at the University of Greifswald for a routine dental check-up, accompanied by her parents.

**Clinical findings**

The patient did not report any symptoms or complaints. During the dental examination, poor oral hygiene and a history of consuming sugary foods and drinks were documented. Dental history revealed that the patient had worn a fixed orthodontic appliance for 18 months, which might have negatively affected the patient’s oral hygiene and caries activity. The clinical examination (11/2019) revealed a suspicion of multiple proximal carious lesions.

**Diagnostic assessments**

**Figure 1:**
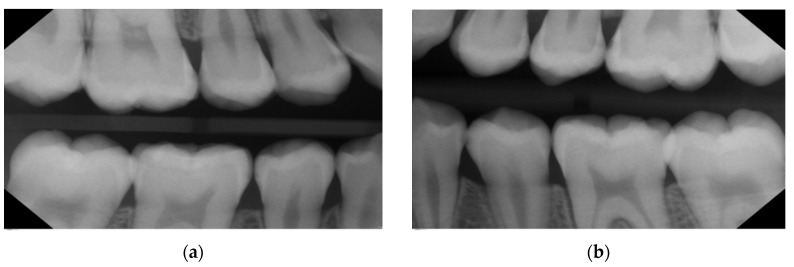
Case 1—Baseline in 11/2019: Bitewings show multiple proximal lesions but without clear cavitation on the right (**a**) and left side (**b**). The patient was 13 years old and had a history of fixed orthodontic appliances. Only non-invasive caries management options were undertaken, as due to COVID-19 pandemic, the patient did not show up for micro-invasive or restorative treatment until 2022—see Figure 2. For a Lesion assessment, see Table 1.

**Figure 2:**
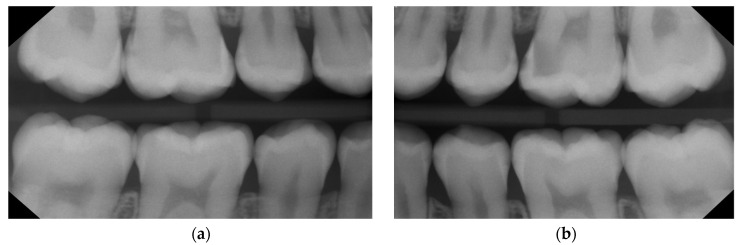
Case 1—After more than 2 years, the bitewings on the right (**a**) and left side (**b**) in 02/2022 show caries progression with a solely non-invasive caries management approach, like instructions to floss and apply fluoride varnish, before the COVID-19 pandemic. The patient is now 15 years old, and the bitewings depict the status before the decision to apply SF on the initial/non-cavitated lesions alongside the restorative treatment (composite restorations in moderate lesions, and for the deep lesion in tooth 26, selective caries removal with the application of biodentine (Septodont) prior to the restoration); see Figure 3; for a lesion assessment, see Table 1.

**Figure 3:**
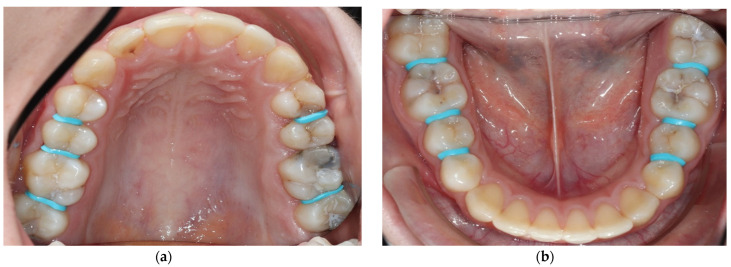
Clinical photos after placement of orthodontic separators proximally but prior to SF application (04/2022, patient’s age: 15 years) in the upper (**a**) and lower jaw (**b**). Tooth 26 was already treated restoratively occluso-mesially with selective caries removal and indirect pulp capping with biodentine. The black staining in 26 occurred due to SF application on the distal lesion of 25 after tooth preparation and biodentine application but before composite filling in 26, as the D1 lesion in 25 was distally clinically non-cavitated (similar to 24).

1. **Clinical diagnostics:** Proximal surfaces were screened with a near-infrared imaging system and revealed the presence of both enamel and dentine lesions.
2. **Bitewings radiographs:** During the same dental visit, bitewing X-rays were taken to confirm the presence of lesions and to determine their depth and proximity to the pulp (Figure 1). The ADA proximal caries classification system was used to diagnose and monitor progression of these lesions: E1: lesion in the outer half of the enamel; E2: lesion within the inner half of the enamel; D1: lesion passing the enamel dentin junction (EDJ) and within the outer third of dentin; D2: lesion within the 2nd third of the dentine; D3: deep lesion passing the 2nd third of the dentin [16]. For evaluations of the bitewing X-rays, three calibrated pediatric dentists classified the lesions independently in a dark room with the option to digitally modify the contrast and brightness of the X-rays. In the rare cases of disagreement, consent was reached via a discussion (Table 1). Bitewings were taken using a Sirona Heliodent DS and a Xios XG supreme intraoral sensor, with a standard dose of 0.16 mAs. The same criteria for bitewings were considered also for the follow-up visit which was at the same time the pre-SDF radiographic examination (Figure 2).
3. **Tooth separation with orthodontic rubbers:** This revealed the absence of cavitation (Figure 3a,b).

These methods were used together as it was found that a combination of all three methods could improve the number of carious lesions detected [17].

In total, 15 initial (non-cavitated) lesions were detected radiographically (02/2022): 9 enamel lesions (E1, E2) and 6 (D1) dentine lesions (Figure 2). Moreover, the high plaque and gingival bleeding index of the patient indicated that the proximal lesions were very likely active [16,18].

**Therapeutic intervention**

1. **Prophylaxis program**

Within the preventive concept in the department, during the same visit, a non-operative approach was taken to control the initial caries lesions. This approach included providing the patient with oral hygiene instructions, diet counselling, dental floss instructions and fluoride varnish every three months to arrest the progression of the lesions, as she was considered to be a high-caries-risk patient.

A disclosing solution was used to show the dental plaque for both the child and their parents. Then, the child was asked to brush their teeth alone with the use of a normal toothbrush with a fluoridated toothpaste to see if this child was brushing his teeth correctly. After that, the child had their teeth cleaned by the dentist using an electric toothbrush attached to a low-speed handpiece. Unfortunately, this was not sufficient, as indicated by the following clinical and radiographical examinations. Likely due to the COVID-19 pandemic and its restrictions, the patient neither followed the recommendations of regular follow-up nor of micro-invasive and/or restorative approaches, for instance, for the teeth 14, 24 and 26.

2. **Indicated use of SF**

After a long time span of 27 months from the first bitewings (11/2019), new bitewing X-rays were taken (02/2022), which revealed, in line with the clinical findings, a clear progression of the existing proximal lesions and development of new lesions. This highlights the caries activity of the patient (Figure 2). The stage and location of the proximal lesions are listed in detail (Table 1). Micro-invasive treatment options such as resin infiltration were recommended for management of the non-cavitated lesions (E1, E2, D1) and restoration of the moderate to deep lesions. In contrast to the first visit, the use of silver fluoride products had tremendously increased in the department due to the COVID-19 pandemic. Therefore, this time, the novel “experimental” application method with SF presented in this article was also offered. The patient and parents were provided with a detailed explanation of the treatment options, including their advantages, disadvantages, time and cost. Following the discussion, it was agreed to apply SF (riva star, SDI) to all proximal areas with initial proximal lesions. Composite restorations were planned for moderate lesions, and for the deep lesion in tooth 26, selective caries removal with the application of biodentine (Septodont) prior the restoration was performed, and silver fluoride was applied directly on the distal surface of 25 in case no cavitation was present during the restorative session for tooth 26.

**Treatment steps of for managing active initial/likely non-cavitated proximal caries lesions**

(1) **Placement of orthodontic separators**

**Figure 4:**
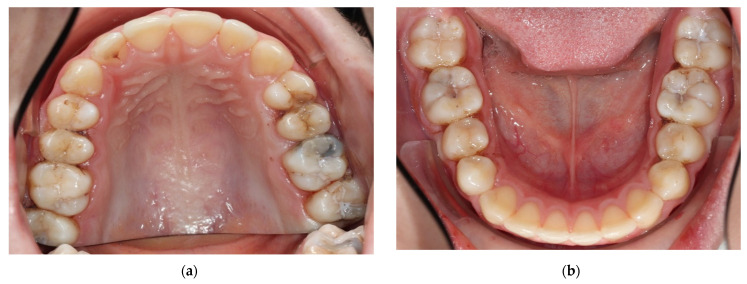
Clinical photos in 04/2022 directly after application of SF (riva star, SDI) in upper (**a**) and lower jaw (**b**); same day as application of separators for 2 h; patient’s age: 15 years. Partial irritation and black staining of the gums can be seen interproximally, which usually disappears within a few days and does not cause long-term effects as the follow-up photos (see Figure 5) demonstrate.

Separators were placed in all approximal areas with initial proximal caries lesions for two hours to facilitate direct clinical inspection and direct application of SF using a micro-brush (Figure 3). After removing the separators, a short time span of approximately 5–10 s was used for direct clinical examination. Then, SF was applied quickly with a small micro-brush was used in the proximal areas (Figure 4). It is important to note that the proximal contact area was not always fully open to allow complete insertion of the micro-brush in all surfaces over the entire application time. Still, the prior tooth separation improved accessibility for an initial short clinical examination and facilitated the flow of the SF to the lesion. In case of a clear sign of a manifest cavitation, restoration would have been planned.

(2) **SF application (Riva Star^®^, SDI)**
  ▪ Petroleum gel was used to protect and avoid/reduce staining of lips and surrounding extra-oral soft tissue [19].
  ▪ Other tooth surfaces were isolated using cotton rolls and a saliva ejector to minimize unwanted staining or irritation of soft tissue or other surfaces.
  ▪ Using air, the area was dried before application of the material.
  ▪ SF was applied using a micro-brush for about 30 s to one minute per proximal area [20].
  ▪ A light curing of 10 s for each proximal space was used to accelerate activation of SF and to allow SF to penetrate deeper into the lesion [21].
  ▪ Fluoride varnish (Duraphat, 22.600 ppm) was applied on top of the area to keep the SF in contact with the caries lesion or high-risk surface for as long as possible to prevent saliva from diluting the SF, and most importantly to mask the ammonia taste from the SF product [22,23].

**Follow-up for re-evaluation of the single-time SF application**

**Figure 5:**
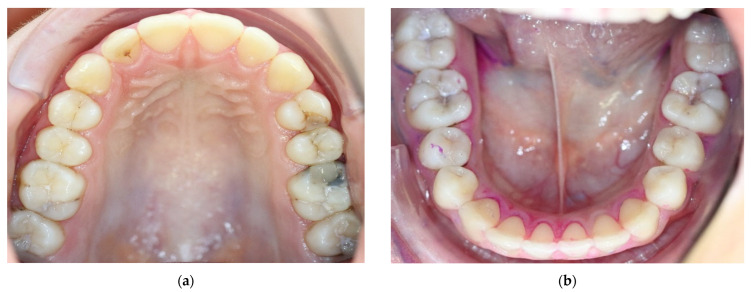
One-year follow-up after SF in 06/2023; patient’s age: 17 years. Clinical photos after staining the plaque and self-brushing of the patient. Composite restorations on 24 and 26 unfortunately still show the discolorations due to SF application on neighboring teeth during the same treatment session (**a**). The staining of the other initial proximal lesions is not or is barely visible and does not cause aesthetic concerns (**b**). Possibly a re-application of SF to the proximal lesions should be considered.

**Figure 6:**
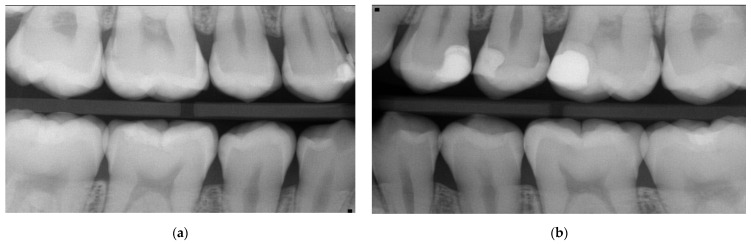
Bitewings on the right (**a**) and left side (**b**) 16 months after SF application show the stages of proximal lesions as well as the integrity of the restorative procedures (06/2023, age: 17 years old) indicating a clear reduction in caries activity and stability of the lesions. For a lesions assessment, see Table 1.

The patient was followed up clinically every 4 to 6 months using the standard prophylaxis program (Figure 5). After 16 months from the last bitewing, another bitewing was planned to assess the progression of the existing lesions and the development of new lesions. The radiographs revealed that there was no evidence of development of new caries lesions. All enamel lesions were stable and did not show signs of caries progression. Among the D1 lesions, one out of six lesions showed signs of cavitation clinically, and required restoration. None of the other dentin lesions displayed radiographic evidence of caries progression (Figure 6 and Table 2).

**Case 2**

**Patient information**

A 13-year-old male patient had been attending the clinic for dental check-ups for approximately seven years. The patient had a medical history of neurodermatitis. His dental history indicated a poor oral hygiene, an uncontrolled diet, and high caries experience in primary molars, most of which were treated in the department to which he was initially referred due to his low cooperative behavior with the family dentist.

**Clinical findings**

The patient’s average proximal plaque index was ~60%, and the gingival bleeding index was ~30%. Despite receiving instructions to improve his oral hygiene and diet, including the recommendation to use fluoride gel (12,600 ppm) once a week at home and dental floss (although it was likely not consistently used), the presence of smooth surface initial caries and a high-caries-risk for the patient remained evident.

**Diagnostic assessments**

**Figure 7:**
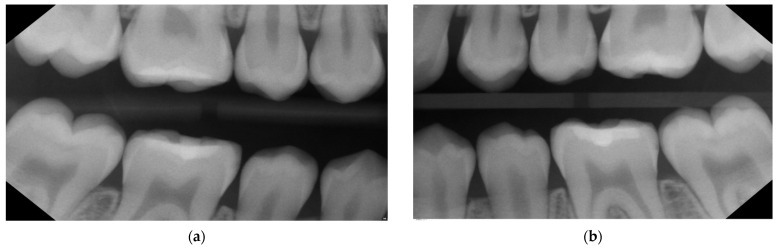
Case 2—Baseline: bitewings in 2019 show the proximal non-cavitated lesions on the right (**a**) and the left side (**b**) at the age of 13. Only non-invasive caries management options including regular recall and fluoride varnish application were undertaken at this point. For a lesion assessment, see Table 2.

Diagnosis was made using the same diagnostic tools as in the first patient. Bitewing reading showed multiple initial caries lesions on posterior teeth but no clearly cavitated proximal lesion (Figure 7 and Table 2). In total, 21 initial proximal lesions were detected: 16 enamel lesions (E1, E2) and 5 dentine lesions (D1). The lesions were more likely to be active according to the high plaque and gingival bleeding index.

**Therapeutic intervention**

1. **Prophylaxis program**

The patient was given instructions on oral hygiene and the use of dental floss regularly, in addition to the prophylaxis program at the dental department, which was the same as for the first patient. The patient was scheduled for application of fluoride varnish every 3–4 months. In contrast to case report one, this patient attended all of his appointments regularly, except for only one missed appointment during the COVID-19 pandemic lockdown. Despite regular appointments and instructions, the patient’s oral hygiene had barely improved and the sugary drinks were still consumed on a regular basis.

**Indicated use of SF and treatment steps**

**Figure 8:**
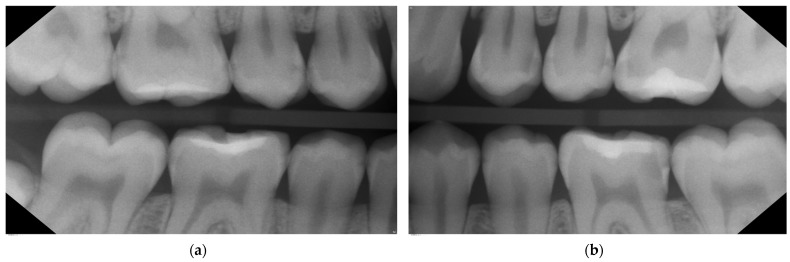
Case 2—Bitewings on the right (**a**) and left side (**b**) after slightly more than three years in 2022 show the progression and development of new proximal caries lesions. At this stage, at the age of almost 16, the decision was taken to apply SF proximally; 36 was treated with a composite restoration due to a clinically assessed cavitation. For a lesion assessment, see Table 2.

**Figure 9:**
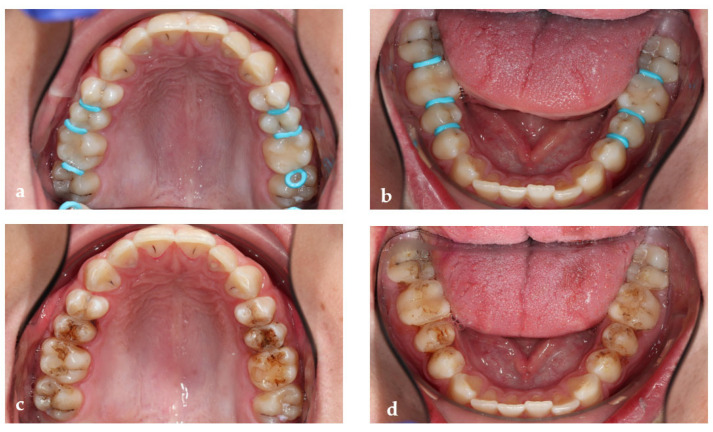
Clinical photos in 2022 (patient’s age is almost 16) after applying the orthodontic separators proximally in the upper (**a**) and lower jaw (**b**) and immediately after SF application and light curing (**c**,**d**). The black staining on the healthy enamel will disappear with brushing at home or can also be brushed away with a polishing paste in the office.

After approximately three years from the first bitewings, new bitewings were taken, which revealed the presence of new caries lesions and progression of the previous proximal lesions, showing that the high caries risk recall program was not sufficient (Figure 8). The stages and locations of the initial proximal lesions are listed in detail (Table 2). After discussing the various alternatives with the parents and the child, taking into account the cost, time and feasibility, it was decided to use SF for all initial lesions and composite fillings for moderate lesions with cavitation (which are paid for by health insurance within the German reimbursement system until the age of 15). For D1 lesions, as they were clinically not detectable at all, it was agreed to place separators for a better assessment of surface integrity (Figure 9a,b). The patient was to be followed up regularly, and if cavitation was present, fillings would be performed. Separators and SDF were applied to a total of 19 initial lesions using the same procedure as in the first patient (Figure 9a,b), and the patient was scheduled for follow-up about every 3 months with fluoride varnish application. The clinical photos show, in addition to what was depicted in case one, that sometimes not all separators remained in their spot even after only two hours (Figure 9a,b). Unfortunately, the more separators are applied in one quadrant, the less space is gained proximally and the less time there is to investigate the proximal surfaces and apply the SF. Furthermore, this illustrates the black staining on the healthy enamel after SF application and light curing. This staining will disappear with brushing at home or can also be brushed away with a polishing paste in the office (Figure 9c,d).

**Follow-up for re-evaluation of SF**

**Figure 10:**
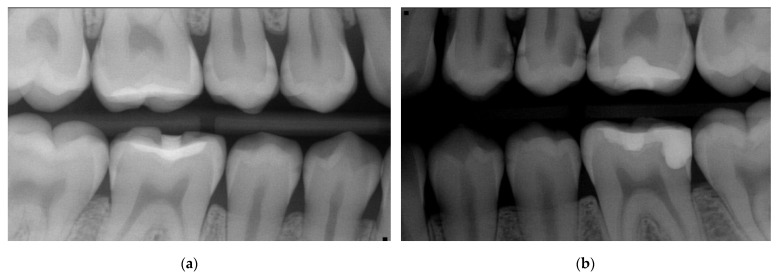
Case 2—Bitewings (03/2023) on the right (**a**) and left side (**b**) at the 1-year follow-up after SF application. The patient’s age is almost 17. The radiographic situation shows both the progression (only in second quadrant (**b**)) and stagnation of (both enamel and D1) proximal lesions after single application of SF within regular recall visits.

In March 2023, one year after the last bitewing, new bitewing radiographs were taken to assess the progression of the existing lesions, with a specific focus on the D1 lesions. The radiographs revealed that there was no evidence for development of new caries lesions. All enamel lesions were stable and did not show signs of caries progression. Among D1 lesions, only one out of five lesions showed caries progression regarding the classification system (Table 2), but some other dentine lesions progressed slightly within their category (e.g., D1 or D2) and restorative measures were planned (Figure 10). Interestingly, caries progression was only observed in the second quadrant, so despite the tooth separation, micro-cavitation possibly remained undetected due to the short examination time or due to the fact that SF could not be sufficiently applied to these lesions.

## 3. Discussion

The main focus in managing initial caries lesions should be on non-operative treatment, aiming to avoid or delay restorative measures by enhancing remineralization to arrest lesion progression, or at best, reverse it. Though it is well evident that approximal initial caries can be treated non-operatively, as lesions confined to the EDJ are most likely non-cavitated (only 8–19% are cavitated), most dentists tend to intervene surgically in such lesions in permanent teeth. This could be concerning, as dental restorations have a limited lifespan; thus, initiating a restorative cycle will eventually compromise the integrity and survival of the tooth [24,25,26].

To best implement minimal intervention measures, early diagnosis is the primary objective [27]. Conventional bitewings remain the gold standard to diagnose and detect approximal lesions, though they have a low sensitivity for the detection of early lesions extending only to the outer enamel, this ranges from 51 to 64% for enamel lesions and is 67% for caries reaching the enamel–dentin junction (EDJ) [28,29]. This means that 33% of lesions confined to the EDJ may go undiagnosed. The sensitivity is probably even lower in real-world settings, where the quality of the radiographs is not always optimal. To enhance the standardization of lesion monitoring and ensure consistent radiation direction, the utilization of individualized bitewing holders would be a valuable additional tool. Therefore, it is advisable to use bitewings in conjunction with other diagnostic tools (as, e.g., tooth separation for direct clinical examination, NIR or FOTI) for a comprehensive assessment.

Nonetheless, in the context of monitoring lesions, bitewing radiography offers a suitable sensitivity and specificity for early detection without a significantly increased risk of false positives. Moreover, bitewings become increasingly sensitive as lesions progress, which enhances their effectiveness in assessing progressed lesions compared to their initial baseline assessment. Still, irrespective of the treatment performed, dentists should be cautious in diagnosing non-progression from enamel to EDJ lesions, as a certain risk of underestimation remains due to the described diagnostic limitations.

In Germany, this is typically recommended to be performed at intervals determined by the patient’s caries risk. Bitewings in high-risk children should be considered about every 12–24 months [30].

SF has been proven to be highly effective in preventing caries development and arresting ~80% of dentine carious lesions in high-caries risk patients [31,32]. Moreover, unlike other micro-invasive techniques, which create a mechanical barrier against the biofilm [33], SF works in a biological way, as the distinctive anti-microbial activity and high fluoride content not only arrest caries, but also promote remineralization [21]. This has been demonstrated in retrospective and in vitro studies [34].

This case series demonstrated that SF was highly effective in preventing the progression of enamel carious lesions (E1 and E2), as no evidence of progression was observed even in highly caries active adolescents. However, SF was less effective in dentin lesions (D1), with still only two out of eleven lesions showing progression and requiring restorative treatment. The reduced efficacy of SF in D1 lesions may be attributed to a higher bacterial load and deeper bacterial penetration into the dentin. These micro-cavitations are difficult to clean, can protect the biofilm and consequently facilitate the progression of caries [30,35]. This could be due to an incorrect clinical baseline assessment (cavitation not seen), insufficient use of the micro-brush to apply the SF or persisting caries activity in the patient due to, e.g., irregular home use of dental floss, insufficient fluoride uses and/or a high frequency of sugar intake.

In line with the existing literature, these cases show that non-invasive approaches, including fluoridated toothpaste, flossing and fluoride products, proved insufficient to arrest caries progression or to prevent its development in high-caries-risk patients. Although dental flossing is a suitable method for mechanical plaque removal from proximal surfaces [36,37], it is not widely practiced among the general population [9]. Therefore, the effectiveness of this approach relies largely on patient compliance, which may lead to an insufficient level of lesion management [38,39,40].

Micro-invasive approaches, such as proximal sealants and resin infiltration, demonstrate a high amount of evidence in reducing the risk of caries progression. However, they are time-consuming and more expensive. For instance, the time required to place proximal sealants or infiltrants is roughly comparable to the time required for a two-surface composite filling. Moreover, these treatment options are technique-sensitive for both patients and operators [41,42,43]. This complexity is further compounded when multiple surfaces are involved.

SF application was demonstrated to be easy, simple, quick and time-efficient in the sense that it allows for the management of multiple surfaces at a time. Despite involving a two-visit dental procedure, the procedural time on the dental chair is relatively short, especially when compared to treatment alternatives (e.g., caries infiltration) for a high number of lesions. Additionally, the use of SF-soaked superfloss could always be considered to eliminate the need for an additional appointment for separator placement when not feasible, but this comes with the drawback of lacking direct clinical assessment. Furthermore, SF is highly cost-efficient, accessible and equitable for all socio-economic groups, making it a valuable option not only in Europe but also worldwide.

Caries arrest using SF is considered a safe treatment, and it rarely causes any local symptoms such as pain or gum swelling [44]. The only apparent drawback of SF is the dark staining of the lesions, and acceptance of the staining varies with different cultures and the affected teeth or surface. However, in general, the acceptance of the staining in posterior teeth is far better than in anterior teeth [45]. More importantly, as demonstrated in this case series, black staining of the (arrested) proximal lesions caused by SF was hardly visible due to the tight contact surfaces of permanent teeth (Figure 4 and Figure 5), but staining of composite fillings may occur when applied in the same session before the restoration itself.

We want to highlight that this approach is highly innovative and has not been reported in the literature. It has demonstrated high effectiveness for managing initial enamel proximal lesions. However, for dentine lesions, it may be advisable to consider multiple applications, possibly in the 3- or 6-month follow-up visits.

We strongly emphasize the importance of conducting different types of clinical research in this area to establish evidence for the effectiveness of SF application in managing initial proximal carious lesions using various techniques, preferably RCTs comparing the efficacy of SF with, e.g., other fluoride varnishes, caries infiltration or self-assembling peptides, should be taken into consideration.

## 4. Conclusions

The application of 38% silver fluoride after tooth separation with orthodontic rubbers is a cheap, easy and quick method to directly diagnose caries and arrest multiple proximal active non-cavitated enamel lesions at a time in permanent teeth, while dark staining of these lesions is not or is barely visible in clinical follow-up examinations. For proximal dentin lesions without obvious cavitation, a re-application of SF may be useful, or other micro-invasive or traditional approaches may be necessary.

## 5. Patients’ Perspective

**Case 1**

This patient was very surprised and disappointed that we found so many proximal lesions after the COVID-19 pandemic, as she did not have symptoms and was not expecting this at all in the control visit in February 2022. After experiencing local anesthesia and receiving fillings following this diagnostic visit, she and her mother were very satisfied that we could reduce the number of invasive treatments, and consequently the number of necessary dental visits, via application of SF.

**Case 2**

This patient did not care so much about the fact that he had caries and whether this meant that he might receive fillings or not, but rather cared about making multiple visits for their treatment. He and his mother were, therefore, highly satisfied with the technique, as we mainly could reduce the number of necessary dental visits. As they have quite a long drive to reach the clinic, irrespective of the duration of the dental visit, at least half a day is spent on attending treatment sessions. Despite the fact that some dentin lesions progressed (second quadrant), they were happy that invasive treatment was postponed.

## Figures and Tables

**Table 1:** Stages of carious lesions radiographically for case 1 at baseline 11/2019 (pre-pandemic); in 02/2022 (before SF application and restorative treatment); and 1.5 years later in 06/2023 as follow-up after the SF application in both upper and lower permanent teeth for this highly caries active adolescent patient, documented by caries progression in the first years (from 2019 to 2022) and stability from after SF application and restorative treatment (from 2022 to 2023).

Tooth	17	16		15		14		24		25		26		27
Surface	M	D	M	D	M	D	M	M	D	M	D	M	D	M
**Figure 1** **—11/2019**	?	0	E1	E2	E2	E2	D1	0	D1	E2	D1	D2	0	0
**Figure 2** **—02/2022**	?	D1	D1	D1	E2	D1	-	-	D2	D1	D1	D3	E2	?
**Figure 6—07/2023**	?	D1	D1	D1	E2	D1	F	0	F	F	D1	F	E2	0
**Tooth**	**47**	**46**		**45**		**44**		**34**		**35**		**36**		**37**
**Surface**	**M**	**D**	**M**	**D**	**M**	**D**		**D**		**M**	**D**	**M**	**D**	**M**
**Figure 1** **—11/2019**	0	0	E1	E1	0	0		E2		E1	E2	0	0	?
**Figure 2** **—02/2022**	0	0	E1	E2	0	0		E2		E1	E2	E1	E1	?
**Figure 6—07/2023**	0	0	E1	E2	0	0		E2		E1	E2	E1	?	0

(?) = cannot be assessed, (-) = not included in the X-ray, (F) = filling.

**Table 2:** Case 2: Stages of the carious lesions radiographically at baseline in 2019 in the upper jaw and lower jaw, just before SF application in 2022 and 1 year later in 2023 in both upper and lower permanent teeth for this highly caries active adolescent patient documented by the caries progression from 2019 to 2022.

Tooth	17	16		15		14		24		25		26		27
Surface	M	D	M	D	M	D	M	M	D	M	D	M	D	M
**Figure 7** **—02/2019**	0	0	E1	0	0	0	0	0	0	0	E1	D1	0	0
**Figure 8** **—03/2022**	E1	D1	E1	E2	D1	E2	0	0	D2	D1	D1	D2	E1	E1
**Figure 10—03/2023**	E1	D1	E1	E2	D1	E2	0	0	D2	D1	D3	D2	E1	0
**Tooth**	**47**	**46**		**45**		**44**		**34**		**35**		**36**		**37**
**Surface**	**M**	**D**	**M**	**D**	**M**	**D**		**D**		**M**	**D**	**M**	**D**	**M**
**Figure 7** **—02/2019**	0	0	0	0	0	0		0		0	0	0	E1	0
**Figure 8** **—03/2022**	E1	D1	E1	E1	E2	E1		E1		E2	E1	E1	D2	E1
**Figure 10—03/2023**	E1	D1	E1	E1	E2	E1		E1		E2	E1	E1	F	E1

(?)= cannot be assessed, (-) = not included on the X-ray, (F) = filling.

## Data Availability

Data are fully presented in the article.
